# Mosquitoes infected with dengue viruses in Brazil

**DOI:** 10.1186/1743-422X-7-152

**Published:** 2010-07-12

**Authors:** Mario LG de Figueiredo, Almério de C Gomes, Alberto A Amarilla, André de S Leandro, Agnaldo de S Orrico, Renato F de Araujo, Jesuína do SM Castro, Edison L Durigon, Victor H Aquino, Luiz TM Figueiredo

**Affiliations:** 1 Institute of Biomedical Sciences of the University of Sao Paulo, Sao Paulo, Brazil; 2 School of Public Health of the University of Sao Paulo, Sao Paulo, Brazil; 3 Virology Research Center, School of Medicine of Ribeirao Preto, University of Sao Paulo, Ribeirao Preto, Brazil; 4 Zoonosis Center of the City of Foz do Iguaçu, PR, Brazil; 5 Ministry of Health of the State of Bahia, Salvador, BH, Brazil; 6 Department of Clinical, Toxicological and Bromatological Analysis, School of Pharmaceutical Sciences of Ribeirao Preto, University of Sao Paulo, Ribeirao Preto, Brazil

## Abstract

Dengue epidemics have been reported in Brazil since 1985. The scenery has worsened in the last decade because several serotypes are circulating and producing a hyper-endemic situation, with an increase of DHF/DSS cases as well as the number of fatalities. Herein, we report dengue virus surveillance in mosquitoes using a *Flavivirus *genus-specific RT-Hemi-Nested-PCR assay. The mosquitoes (*Culicidae*, n = 1700) collected in the Northeast, Southeast and South of Brazil, between 1999 and 2005, were grouped into 154 pools. Putative genomes of DENV-1, -2 and -3 were detected in 6 mosquito pools (3.8%). One amplicon of putative DENV-1 was detected in a pool of *Haemagogus leucocelaenus *suggesting that this virus could be involved in a sylvatic cycle. DENV-3 was found infecting 3 pools of larvae of *Aedes albopictus *and the nucleotide sequence of one of these viruses was identified as DENV-3 of genotype III, phylogenetically related to other DENV-3 isolated in Brazil. This is the first report of a nucleotide sequence of DENV-3 from larvae of *Aedes albopictus*.

## Findings

Dengue viruses (family *Flaviviridae*, genus *Flavivirus*), serotypes 1 to 4 (DENV-1, -2, -3 and -4), are responsible for large urban outbreaks. Infection with any of the four dengue virus serotypes can lead to acute febrile illness and to the severe, sometimes fatal, dengue hemorrhagic fever/dengue shock syndrome (DHF/DSS) [1].

DENV are transmitted to humans mainly by *Aedes aegypti *mosquitoes, which acquired the infection through blood-feeding on infected individuals or by transovarial transmission [2]. Besides, in Africa and Asia, DENV have been reported in sylvatic (enzootic) cycle involving non-human primates and various species of *Aedes *mosquito (such as *Ae. furcifer*, *Ae. luteocephalus *and *Ae. taylori*) [3].

The first viral isolation in Brazil was reported in 1981 in Roraima Sate, Northern Brazil, where DENV-1 and DENV-4 were isolated and associated with dengue cases. DENV-1 and DENV-2 were introduced in Rio de Janeiro, in 1985 and 1990, respectively; then, both viruses co-circulate for 10 years causing several outbreaks in the country, including many cases of dengue hemorrhagic fever. In 2000, DENV-3 was introduced in Rio de Janeiro State and then spread to all the country, co-circulating with DENV-1 and DENV-2. Finally, DENV-4 was isolated from dengue fever cases in Manaus at Amazon State, in 2005, suggesting its circulation in that City [4,5]. Actually, Brazil is facing a hyper-endemic situation with increase number of DHF/DSS in children and fatal cases [6,7].

Herein, we report results of dengue virus surveillance in mosquitoes using a RT-Hemi-Nested-PCR assay.

Using light traps in the soil and the top of the trees, 1700 mosquitoes (*Culicidae*, Diptera) were captured in three different places of Brazil (Table 1), where dengue outbreaks have been reported [8]. Thus, in Coribe County (13° 49' 44" S, 44° 27' 14" O), Bahia State, Northeast region, 644 mosquitoes were collected in the rain forest, in 2002. In the City of Foz do Iguaçu (25° 32' 52" S, 54° 25' 16" O), Parana State, South region, 370 mosquitoes were collected at urban area, in 2005. In the City of Santos (23° 56' 13.16" S, 46° 30' 34" O), São Paulo State, Southeast region, 686 mosquitoes were collected in the urban area, in 1999. In addition, larvae were collected from domestic and peridomestic containers.

**Table 1:** Origin and number of *Culicidae *(Diptera) collected for the study.

Collection places	Species	Adults	Larvae	Males	Females	Number of pools
Coribe County	*Psorophora albipes*	50	**-**	**-**	50	5
Coribe County	*Psorophora albigenus*	50	**-**	**-**	50	5
Coribe County	*Psorophora ferox*	60	**-**	**-**	60	6
Coribe County	*Haemagogus jantinomys*	64	**-**	10	54	9
Coribe County	*Haemagogus leucocelaenus*	171	**-**	1	170	21
Coribe County	*Haemagogus spegazzinii*	249	**-**	241	8	21
City of Foz do Iguacu	*Aedes Aegypti*	370	**-**	19	403	51
City of Santos	*Aedes aegypti*	56	**-**	13	43	4
City of Santos	*Aedes albopictus*	88	542	39	49	32 (6 of adults and 26 of larvae)
**Total**		**1158**	**542**	323	887	154

Mosquitoes and larvae were identified in CO_2 _atmosphere, based on morphologic characteristics [9] and those from the same specie or genus, captured in the same place, were pooled (~10 adult or larvae mosquitoes/pool) based on day of collection and stored at - 70°C (Table 1). To each mosquito pool, 1.5 ml of 4% bovine albumin in PBS (pH 7.8) were added. The specimens were crushed using grind and mortar, and centrifuged at 2500 × g, for 30 minutes, at 4°C. The supernatants were split in two aliquots and stored at - 80°C until use [10].

**Figure 1:**
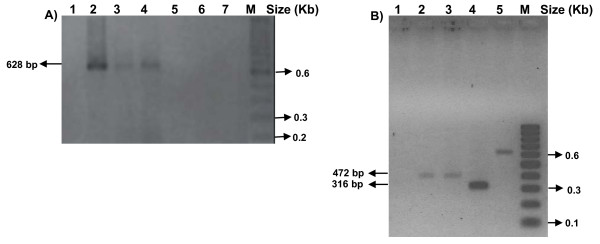
**Agarose gel electrophoresis showing amplicons obtained by the RT-Hemi-Nested-PCR for flavivirus from mosquitoes and larvae**. ***A***) Lanes 1 to 7 include amplification reaction products from larvae of *Aedes albopictus *from the City of Santos. Amplicon of ~628 bp compatible with DENV-3 in lanes 2, 3 and 4. ***B***) Amplicon of ~472 bp, compatible with DENV-1, are shown in lanes 2 (*Aedes aegypti *of the City of Santos) and 3 (*Haemagogus leucocelaenus *from the County of Coribe). Line 4 shows an amplicon band of ~316 bp, compatible with DENV-2, obtained from *Aedes aegypti *captured in Foz do Iguaçu City. Lane 1 is a negative control and lane 5 is a positive control (DENV-3) of the RT-Hemi-Nested-PCR reaction.

RNA from the supernatant of macerated mosquito samples was extracted using the Qiamp Viral RNA Kit (QIAGEN, USA). RNA extracts were subjected to a *Flavivirus *genus-specific RT-Hemi-Nested-PCR that allows the identification of DENV-1 to 4, YFV, ILH, SLEV, BSQV and ROCV [11]. The size of the amplification products suggests the presence of DENV genomes in 6 (3.8%) pools (Figure 1), and the information on theses mosquitoes is summarized in Table 2. Amplicons having DENV-1 compatible size were obtained from a pool of *Haemagogus leucocelaenus *captured in Coribe County and from adult females of *Aedes aegypti *capture in Santos City [11]. One amplicon with DENV-2 compatible size was amplified from a pool of *Aedes aegypti *captured in Foz do Iguaçu City [11]. Finally, DENV-3 compatible amplicon was obtained from 3 pools of larvae of *Aedes aegypti *collected in Santos [11].

**Table 2:** Information on 6 mosquito pools having flavivirus genome amplified by RT-nested-PCR [11].

Collection places	Date of Collection	Genera	Specie	Amplicon size	Virus
Coribe County	**2002**	**Female**	***H. leucocelaenus***	**~472 pb**	**DENV-1**
City of Foz do Iguacu	2005	Female	*Ae. Aegypti*	~316 pb	DENV-2
City of Santos	**1999**	**Larvae**	***Ae. albopictus***	**~628 pb**	**DENV-3**
City of Santos	1999	Larvae	*Ae. albopictus*	~628 pb	DENV-3
City of Santos	1999	Larvae	*Ae. albopictus*	~628 pb	DENV-3
City of Santos	1999	Female	*Ae. Aegypti*	~472 pb	DENV-1

The sequenced amplicon is shown in bold.

**Figure 2:**
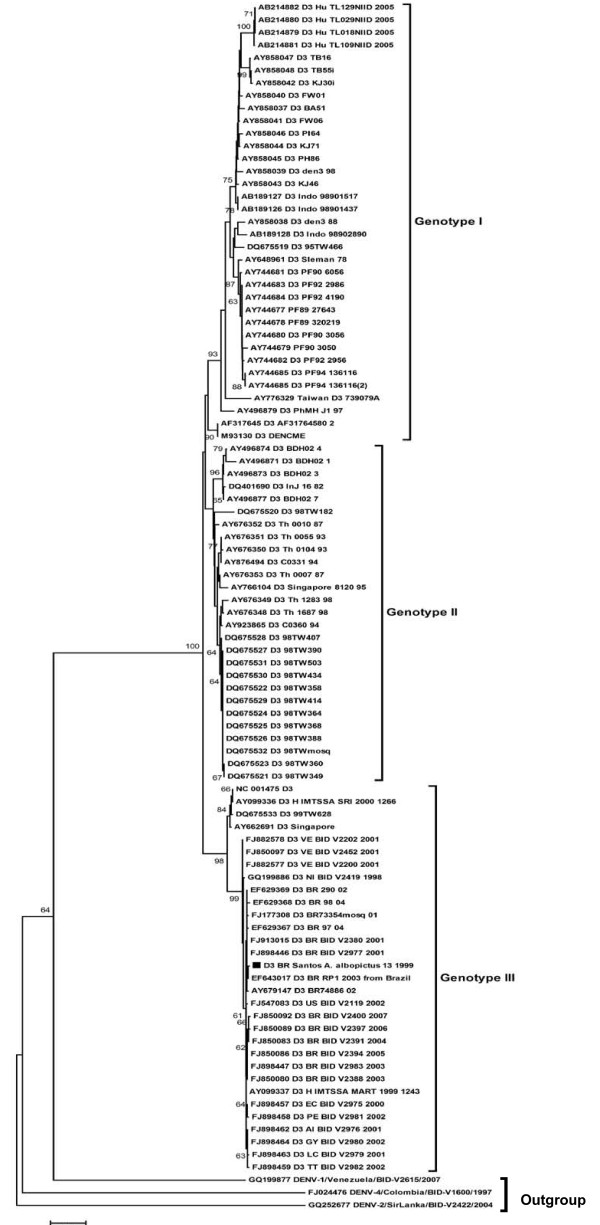
**DENV-3 phylogenetic tree based on the NS5 partial gene sequences**. The three was constructed using the method of Neighbor-joining with 1000 bootstrap replications. The genotypes are labeled according to the scheme of Lanciotti in 1994 [14] and Amarilla in 2009 [15]. DENV-1, DENV-2 and DENV-4 were used as outgroup. Branch lengths are proportional to percentage of divergence. Tamura Nei (TrN+G) nucleotide substitution model was used with a gamma distribution (G) of 0.5121. Bootstrap support values are shown for key nodes only (values < 70% not shown). The strains isolated D3/BR/Santos/A. albopictus 13/1999 is marked with a filled square. The GenBank accession numbers, species, the country of origin, and year of isolation are shown.

The amplicon of DENV-3 obtained from the pool of larvae of *Aedes albopictus *was directly sequenced after purification from the agarose gel with the QUIAquick gel extraction (Qiagen, USA). The purified product was sequenced in an ABI PRISM^®^3100 Genetic Analyzer (Applied Biosystems, Foster City, CA-USA). The obtained 568 base pair sequence, named D3/BR/Santos/A. albopictus 13/1999, was registered in the GenBank with the accession number HM053487. This sequence was aligned with 569 worldwide DENV-3 retrieved from GenBank using the program CLUSTAL W software [12]. The alignment was edited with the software MEGA 4.0 [13]. The phylogenetic relationship among strains was reconstructed by the neighbor-joining (NJ) using MEGA 4.0. The analyses were supported by bootstrap using 1000 replicates. Figure 2 shows the phylogenetic tree with the characteristic distribution of DENV-3 in four genotypes, as previously reported [14,15]. The sequence obtained in this study grouped in the genotype III together with Brazilian strains. The other amplicons could no be sequenced because of the small quantity of the products.

All procedures were performed in order to avoid any type of contamination; different rooms were used for RNA purification, NS5 protein gene amplification and PCR products analysis.

The RT-Hemi-Nested-PCR method used in this study has been shown previously to be a reliable diagnostic tool to detect flavivirus infection in humans [16]. We were able to obtain amplicons of putative DENV-1, DENV-2 and DENV-3, and one of the amplicons was confirmed to be DENV-3 by nucleotide sequencing.

A putative DENV-1 was detected infecting females of *Aedes aegypti *captured in Santos in 1999, at the same time of an outbreak with 4685 reported cases [17].

In addition, a putative DENV-2 was also found infecting the same specie of mosquito captured in Foz do Iguaçu and a putative DENV-1 was detected infecting the same mosquito species from Santos. *Aedes aegypti*, an anthropofilic and urban mosquito, is the most important dengue vector in the Americas and is present in practically all Brazilian cities [18].

Interestedly, we have found putative DENV-1 infecting females of *Haemagogus leucocelaenus *collected in a rain forest of the Northeast of Brazil. This finding might suggest a sylvatic cycle of the virus as previously reported in Africa with DENV-2 and in Asia with DENV-1, -2 and -4, involving non-human primates [3,19]. This may also represent the beginning of sylvatic adaptation of a virus circulating in the urban area. A similar phenomenon has previously occurred with the African YFV, which lead to urban epidemics after its introduction in the Americas, but then, suffered a processes of adaptation to a sylvatic cycle in *Haemagogus janthynomis*, *leucocelaenus *and *Sabethes *spp., and non-human primates [20]. Equally, the sylvatic DENV in Asia is maintained in an enzootic cycle, mainly circulating in canopy-dwelling monkeys, with infrequent spillover to human populations via *Aedes spp*. that feed on both upper and lower canopy primates [21]. Besides, the four serotypes of DENV have been recently reported in French Guyana infecting rodents, marsupials, chiroptera, and showing that sylvatic cycles are occurring in South America [22]. It is known that sylvatic strains of DENV are genetically distinct of the endemic viruses [21]. However, it was not possible to obtain the nucleotide sequence of the amplicon of the putative DENV-1 infecting *Haemagogus leucocelaenus*. Further studies are necessary in order to confirm that DENV-1 is infecting *Haemagogus *in Brasil.

Putative DENV-3 was found infecting 3 pools of *Aedes albopictus *larvae collected in 1999, in Santos City, at the coast of São Paulo, the most populated state of Brazil. The sequence of one of these viruses was identified as DENV-3 of genotype III, phylogenetically related to Brazilian isolates. As far as we know, this is the first nucleotide sequence of DENV-3 ever reported from larvae of *Aedes albopictus*. DENV-3 of genotype III was firstly reported in Brazil in 2000 in Rio de Janeiro [23]. However, based on our data, we can suppose that DENV-3 genotype III was introduced in the Brazilian Southeast coast before 2000. It was also recognized another introduction of DENV-3 genotype III at the North of the country [8]. This finding also suggests that *Aedes albopictus *could have participated as vector in the huge dengue outbreaks occurred in the Brazilian coast. Furthermore, the infection of larvae of *Aedes albopictus *is an evidence of transovarial transmission of DENV-3, as previously reported with DENV-1 [24]. *Aedes albopictus *is a mosquito from Asia that was introduced in Brazil by merchant ships. This mosquito is not as antropophilic as *Aedes aegypti *and can be found in both urban and rural areas [25]. The vertical transmission of DENV ensures presence of the pathogen in mosquitoes independent of their feeding upon an infective human blood carrying DENV. This virus retention across mosquito generations may serve to keep DENV in nature during inter-epidemic periods of the disease being a possible cause of reemergence of dengue in areas previously exposed to the virus. It also may have importance for amplifying an ongoing disease outbreak [26,27].

Laboratory-based mosquito surveillance is important to provide an early warning of dengue fever epidemics, to furnish information on who are the vectors carrying DENV in nature and what is happening in terms of virus transmission during these outbreaks. This knowledge is crucial for vector control measures since we still do not have a dengue vaccine. The RT-nested-PCR used in the present study allowed a fast detection and typing of DENV and other flavivirus in the mosquitoes.

## Competing interests

The authors declare that they have no competing interests.

## Authors' contributions

MLGF, ACG, AAA, VHA and LTMF conceived of the study, and participated in its design and coordination. All authors read and approved the final manuscript.
